# Strain Rate during Creep in High-Pressure Die-Cast AZ91 Magnesium Alloys at Intermediate Temperatures

**DOI:** 10.3390/ma12060872

**Published:** 2019-03-15

**Authors:** Mónica Preciado, Pedro M. Bravo, José Calaf, Daniel Ballorca

**Affiliations:** Escuela Politécnica Superior, University of Burgos, 09006 Burgos, Spain; pmbravo@ubu.es (P.M.B.); josecc33@gmail.com (J.C.); danielballorca@gmail.com (D.B.)

**Keywords:** AZ91, magnesium alloys, creep, high pressure die casting

## Abstract

During creep, magnesium alloys undergo microstructural changes due to temperature and stress. These alterations are associated with the evolution of the present phases at a microstructural level, creating different strain rates during primary and tertiary creep, and with the stability of the inter-metallic phase Mg_17_Al_12_ formed at these temperatures. In this paper, the results of creep testing of high-pressure die-cast AZ91 magnesium alloys are reported. During creep, continuous and discontinuous precipitates grow, which influences creep resistance. The creep mechanism that acts at these intermediate temperatures up to 150 °C is termed dislocation climbing. Finally, the influence of the type of precipitates on the creep behavior of alloys is determined by promoting the formation of continuous precipitates by a short heat treatment prior to creep testing.

## 1. Introduction

The use of magnesium in automotive industry components has increased significantly over recent years [1]. Although the cost of magnesium alloys is disadvantageous in comparison to steel, the weight reduction of the structural component, due to the lower density of magnesium, is highly advantageous. The magnesium alloy AZ91 is one of the most intensively employed magnesium alloys because of its very useful combination of properties such as castability, mechanical performance (at room temperature), corrosion resistance and competitive cost [2]. However, creep resistance begins to yield at temperatures over 127 °C, which limits the use of these alloys to components that are not in major areas [3]. Industrial production of this type of alloy, due to its high productivity and dimensional stability, has primarily been done through high-pressure die casting (HPDC), which introduces porosity in the microstructure [4]. 

One main reason that has been discussed to explain the low creep resistance of the AZ91 alloy is that the microstructure is mainly formed by solid solution α and inter-metallic β-phase (Mg_17_Al_12_) at the grain boundaries, the latter having a low melting point (437 °C). It therefore softens and thickens due to the temperature, resulting in a weakened grain boundary [5]. However, recent hardness tests in the β-phase at temperatures over 200 °C have shown the high deformation resistance of this inter-metallic phase [6], which contradicts the softening theory at the same temperatures.

Creep behavior can, in general, be explained in terms of microstructural stability [7] in connection with hardening mechanisms. Usually, two contradictory trends are observed in creep processes: one is represented by softening processes (cross slip, etc.) and the other by hardening processes (solid solution hardening, precipitation hardening, etc.). When the latter is dominant, creep resistance increases (primary creep), and when the softening processes are dominant, creep resistance decreases (tertiary creep). The minimum creep rate is reached when these two opposing mechanisms reach a balance (steady state or secondary creep) [8]. 

However, during creep tests, high temperatures are reached and this causes the amount of precipitates and their morphology to change. In the literature [9], information has been gathered on the way in which β precipitates, which can be either discontinuous and continuous, and their production is based on how the initial β-eutectic is modified during creep. Of these types of precipitates, it is the continuous precipitates (CP) that have a greater influence on the strength of magnesium alloys [10]. Continuous precipitation consists of alternating acicular-shaped precipitates and is much thinner than discontinuous precipitation [11]. Both types of precipitate coexist and compete in their growth, although there are temperature ranges that favor the growth of one type of precipitation over another. The coexistence of both types is observed at the aging temperature of 150 °C [12], which is the temperature of the creep tests developed in this paper.

The influence of aging treatment on tensile properties in as-cast samples was studied by some authors [8,9], although the time permanence was very high (several hours) and normally given after a solution treatment. In these cases, massive precipitation was formed. In this study, a group of samples was subjected to a very short pre-treatment prior to performing creep tests in order to slightly modify the initial state. The results enabled an evaluation of the role of the developing precipitate types on creep resistance. The studies with aging treatments prior to creep cannot be compared with the results obtained in this paper, as the pre-treatment given to the samples was only 1 h in duration. The purpose of this modification was to create small precipitate nuclei that could favor posterior precipitation in the form of continuous precipitates. 

## 2. Materials and Methods 

The composition of the AZ91D magnesium used in the present study was determined with an Arc Spark Analyzer (wt%): Al, 8.83; Be, 0.001; Cu, 0.007; Fe, 0.003; Mn, 0.32; Si, 0.028 and Zn, 0.6. Differential Scanning Calorimetry (DSC) tests were performed to obtain the temperature at which a phase change occurred, in this case associated with β-phase precipitation from the Al-supersaturated Mg solid solution. Differential Scanning Calorimetry (DSC) tests were performed on a Perkin Elmer DSC7 machine (Waltham, MA, USA) with an argon protective atmosphere. The samples were cut into small discs and heated to temperatures ranging from 30 °C to 230 °C with four different heating ramps: 20, 25, 30 and 40 °C/min. Pure aluminum discs were used as a reference material. 

**Figure 1:**
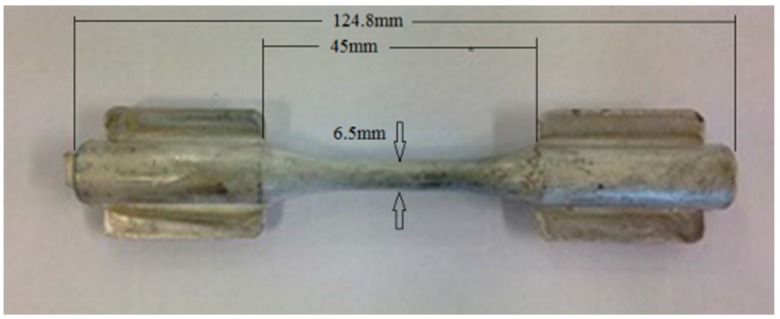
Creep test sample (high-pressure die casting process).

Verification of the precipitate growth in the β-phase was achieved through adiffractometry analysis of the two samples; one was obtained directly from injection and the other by pre-heating at 160 °C for 1 h. The equipment used was a Bruker D8 Advance (Davinci Design, Billerica, MA, USA). The creep tests were performed at constant loads at a temperature of 150 °C with initial stresses of 50, 60, 65 and 70 MPa. For the creep tests, the speed of load application was 1 mm/min and the strains were measured by an extensometer with a gage length of 25 mm. The temperature was maintained in a chamber around the sample and measured by a thermocouple. The specimens used in the creep tests were of circular geometry with a diameter of 6.5 mm. These samples (Figure 1) were obtained by a high-pressure injection process, so that the surface finish and the porosities were the same as the in-service components. These aspects are relevant for the creep behavior. The tests were performed on a Zwick/Roell Kappa 50DS machine (Ulm, Germany). 

From the creep tests, the parameter n, stress exponent, can be calculated from the conventional power law in Equation (1):

(1) $$\varepsilon_{s}={K\sigma }^{n}\exp\left(-\frac{Q_{c}}{\mathrm{RT}}\right)$$

where ε_s_ is the strain rate, K is a constant, Q_c_ is the activation energy, R is the gas constant and T is the temperature. By plotting the minimum ε_s_ versus σ logarithmically, the n exponent is calculated.

The existence of a threshold stress, σ_th_, has been described for precipitate and particle-hardened alloys [13]. If this concept is taken into account, Equation (1) can be modified as:

(2) $$\sigma_{\mathrm{eff}}=\sigma -\sigma_{\mathrm{th}}$$

(3) $$\varepsilon_{s}={k\sigma }_{\mathrm{eff}}^{n_{t}}\exp\left[-\frac{Q_{c}}{\mathrm{RT}}\right]$$

where n_t_ is the true exponent. The precipitates play an important role; however, the samples were not submitted to a solution treatment and most of the Al is in the form of the existing β-eutectic Mg_17_Al_12_ and does not participate in the precipitation produced during creep. As a result, the precipitation is not homogenous and is limited by the existing eutectic β-phase prior to creep. This is why, as estimated by Spigarelli et al. [14], σ_th_ ≈ 0 for die-cast AZ91 alloys. When taking into account the relevant role of precipitates, the n_t_ exponent was calculated according to a method [15] that consists of an extrapolation of logσ − logε_s_ up to ε_s_ = 10^−10^ s^−1^ (considered the lowest measurable creep rate) to obtain σ_th_. It was estimated that the real stress exponent of the HPDC samples should be between conventional, n, and true, n_t,_ exponents.

## 3. Results and Discussion

**Figure 2:**
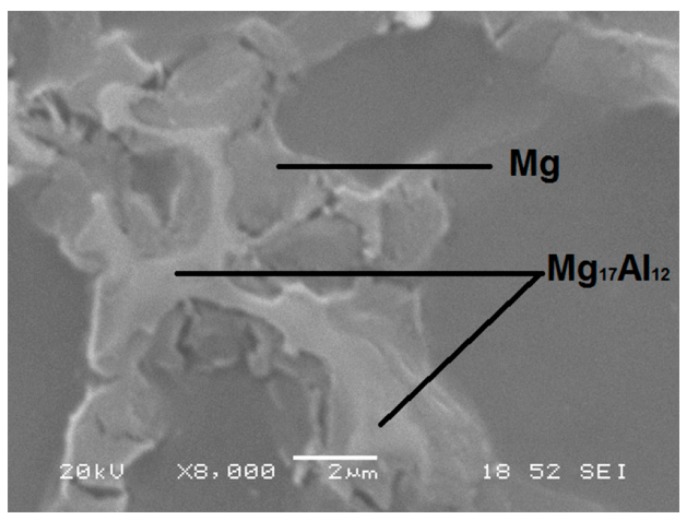
SEM image of divorced eutectic at the grain boundary.

The microstructure of this alloy (Figure 2) consists of Mg-solid solution grains (α-phase) decorated with precipitates in the grain boundary that correspond to a divorced eutectic formed by β-phase (Mg_17_Al_12_) and an Mg-super saturated solid solution (eutectic α-phase). The porosity of the samples is inherent to the manufacturing process and the incompatibility between the hexagonal compact α-phase magnesium matrix and the cubic inter-metallic β-phase, Mg_17_Al_12_, is remarkable.

**Figure 3:**
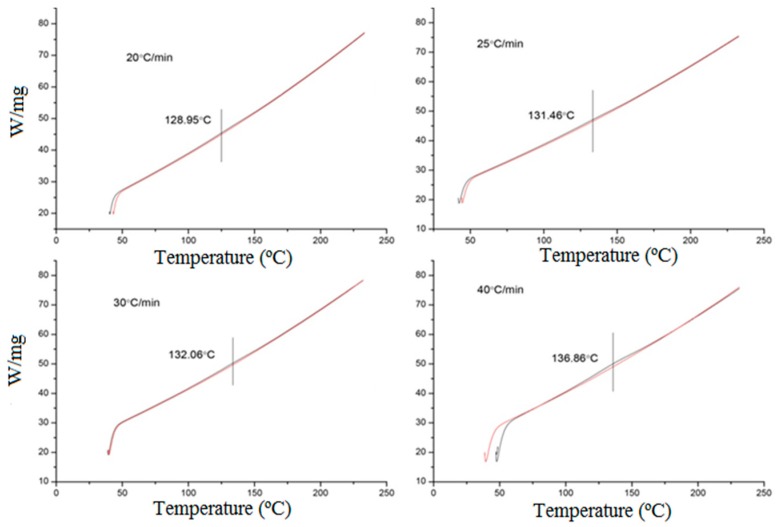
Differential Scanning Calorimetry (DSC) tests at 20 °C/min, 25 °C/min, 30 °C/min and 40 °C/min heating ramps (the vertical line corresponds to the temperature at which a phase change is detected).

The results of the DSC tests are shown in Figure 3. The values at which a small peak was observed were between 129 °C and 137 °C depending on the heating ramp.

**Figure 4:**
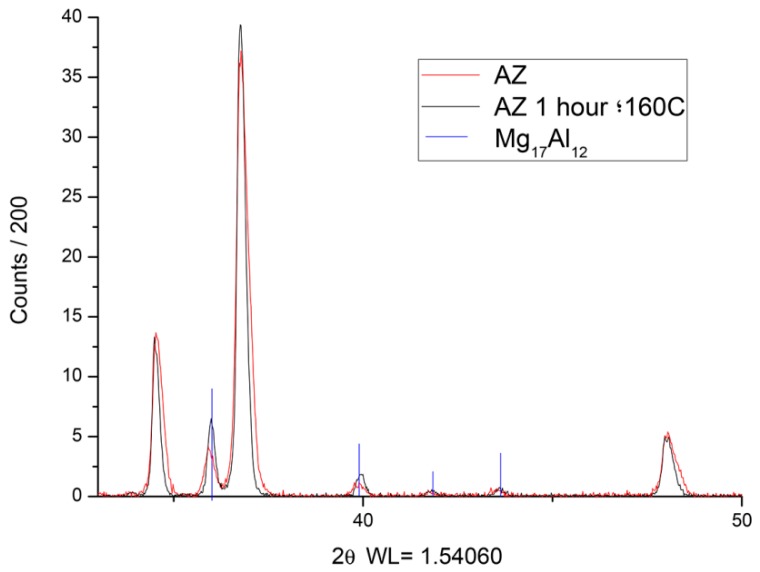
Diffractometry of the as-cast and pre-heated samples. The vertical lines represent Mg_17_Al_12_ location.

The diffractometry of the samples in Figure 4 shows a higher amount of Mg_17_Al_12_ in the pre-treated sample (red line is over the black line).

**Figure 5:**
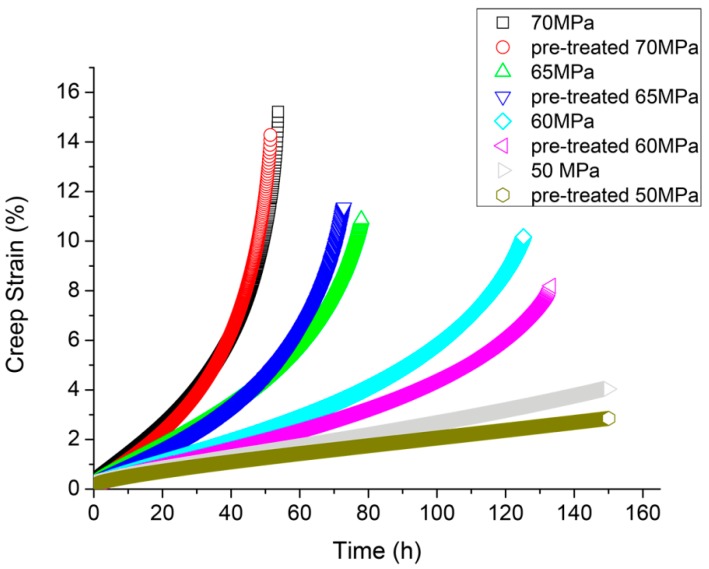
Creep tests in the different samples.

The results of creep are shown in Figure 5. For each condition three samples were tested. The intermediate curve was chosen as the average behavior.

The creep resistance of the pre-treated samples was better for lower loads (50 and 60 MPa). This result is not in contradiction with the results of other authors that clearly showed that aging worsened the creep resistance, because the pre-treatment of 1 h at 160 °C was too short to be considered as aging. 

**Figure 6:**
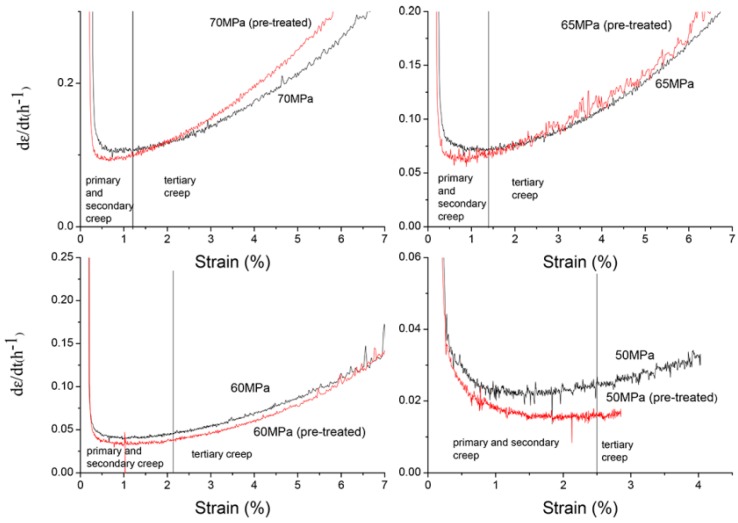
Strain rate variation in creep tests.

To analyze the primary and secondary creep it is convenient to study the curves of strain rate versus strain (Figure 6).

The strain rate during primary and secondary creep was lower for the pre-treated samples although at 65 and 70 MPa the strain rate increased at a higher rate than the non-treated samples once the minimum strain rate was reached. In Figure 6, it can be observed that the pre-treated samples curves were below the non-treated samples during the two first stages of creep at all the loads. At stresses of 65 and 70 MPa a stable secondary creep was not reached (this is more pronounced in the pre-treated samples). A plausible reason for this could be that the precipitates size was small and they are not able to arrest the dislocations movement. When the load decreased to 60 and 50 MPa three stages of creep was observed and the behavior was better for the pre-treated samples at all stages. The stress coefficient and the true stress coefficient was calculated and shown in Table 1. According to the literature (see references in Table 1), the deformation mechanism depended on the value of n. A value of n = 3 was related to the dislocation glide and n = 5 corresponded to dislocation climbing. It is not clear in the literature if the obtained values were based on the values of true n-exponent but in any case, the deformation mechanism seems to be controlled by the climbing of dislocations. 

### Microstructural Analysis

During creep there is precipitation of the intermetallic Mg_12_Al_17_ in the form of continuous and discontinuous precipitates, mostly from the former eutectic Al-supersaturated α-phase [19]. There was also loss of solution hardening with an increase in precipitation hardening in the evolution of the primary creep. It has been stated [20] that a decrease of the creep rate in the primary creep was due to an increase in dislocation density and to the precipitation of β-phase in different forms. At some point these precipitates coarsen with a loss of hardening effect and in some samples a short period of secondary creep was reached. This corresponded to the primary and secondary creep stage as seen in Figure 6. Once the precipitates coarsen and were not able to effectively stop the dislocation movement, the tertiary creep rapidly progressed until rupture. This last stage creep was assisted by the fact that the microstructure is porous. These pores are in the grain boundaries and the incoherence between intermetallic (also in the boundaries) and the matrix helped to make the final fracture.

**Figure 7:**
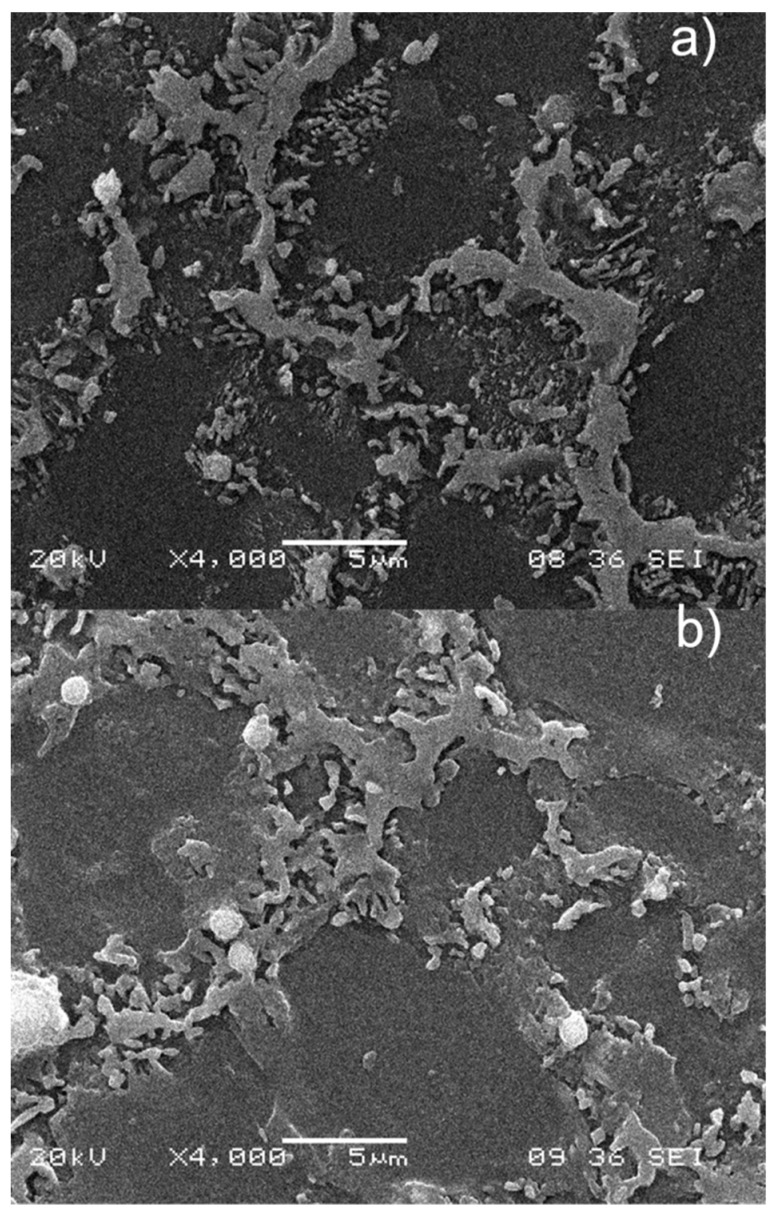
SEM: (**a**) Pre-treated sample after creep test at 70 MPa. (**b**) As-cast sample after creep test at 70 MPa.

**Figure 8:**
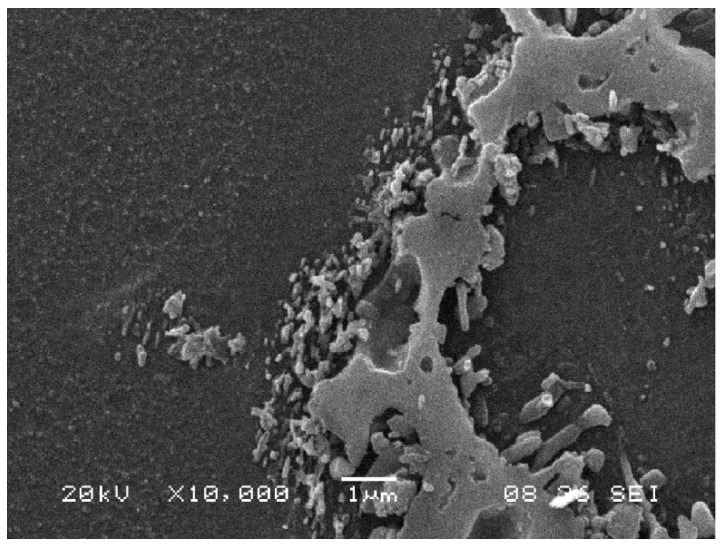
Detail of the precipitates in pre-treated sample at 70 MPa.

SEM (FEI Quanta 600, Hillsboro, OR, USA) observation of the samples after the creep tests showed the microstructure with the precipitates that are formed. In Figure 7, it was observed that the precipitates from eutectic saturated solid solution α decorated both sides of the eutectic β. These samples were loaded at 70 MPa and it was visible that the precipitation was finer in the pre-treated sample. This was observed in greater detail in Figure 8 at a higher magnification.

**Figure 9:**
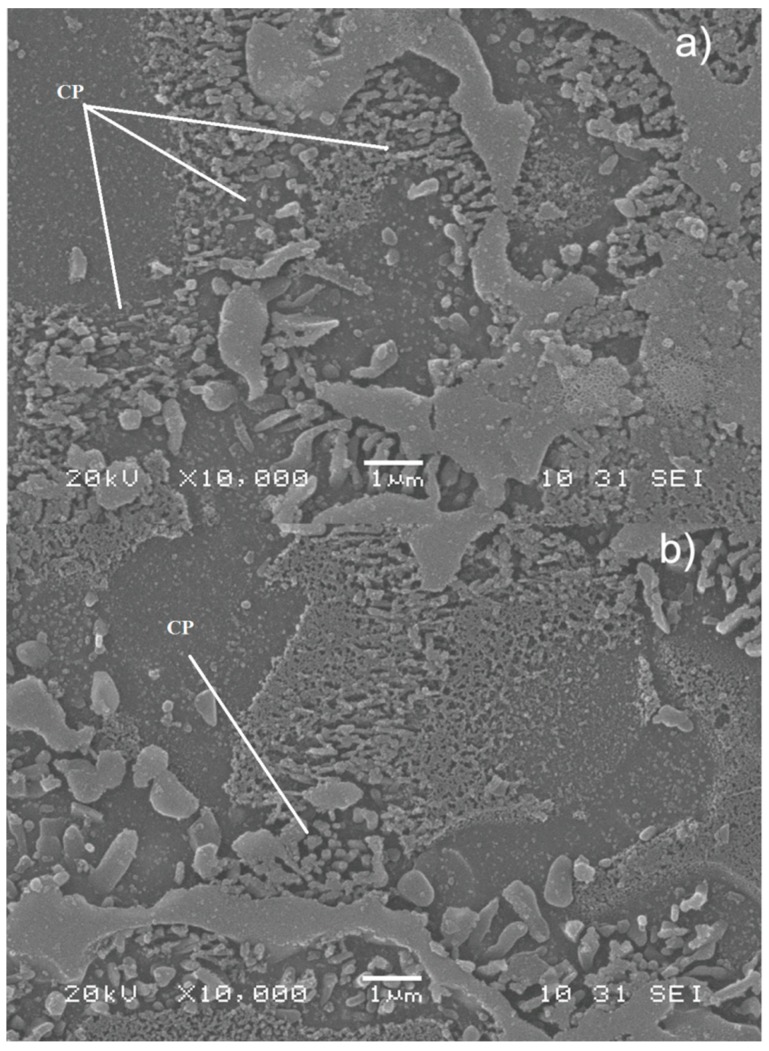
SEM: (**a**) pre-treated sample after creep test at 50 MPa; (**b**) sample after creep test at 50 MPa.

In a comparison of the microstructures at 50 MPa of a pre-treated sample with an as-cast sample (Figure 9), where the maximum difference in strain rate was encountered, the presence of coarsened continuous and discontinuous precipitates was observed in both. It has been noted that the continuous precipitation in the as-cast sample, when encountered, was smaller than that in the pre-treated sample. A plausible reason for this could be the creation of small nucleus sites during the short pre-heating that in the subsequent creep testing lead to continuous precipitates. The absence of continuous precipitation without quenching (after solution annealing) was reported [21], due to the deficiency of nucleation sites. The same study also reported that continuous and discontinuous precipitation occurred when a supersaturated solution was heated. This needs to be taken into account in the present study, where visible SEM β-precipitates appeared in the rich aluminum solid solution zones that were eutectic α before creep. Furthermore, precipitation was assisted by stress, where strain fields resulted in multiple defects that, according to the same author, may act as heterogeneous nucleation sites in the precipitation of continuous β-precipitates. 

On the other hand, continuous precipitation was the most effective means of increasing mechanical resistance to this type of alloy [22]. During primary creep, the higher capacity of strain hardening for the pre-treated samples at all the loads was probably due to the continuous β-precipitates. However, 65 MPa and 70 MPa minimum creep strains were reached almost immediately, and then the softening processes occurred quickly. This suggests that at high stresses the continuous β-precipitates were not effective, probably because of the small size of the precipitates (smaller in the pre-heated samples), which provoked the dislocations to move more easily. At lower stresses the primary creep was longer (40 h for 50 MPa and 17 h for 60 MPa), allowing the precipitates to grow and be more effective in the hardening process. When the precipitates coarsened, the hardening effect decreased and finally the strain rate increased until fracture. The intragranular precipitation of β-phase was not considered in the discussion because the content of aluminum in the primary α was low, so the amount of precipitates would also be low.

The deformation mechanism of dislocation climbing was given by the stress exponent value, which was also higher in the case of the pre-treated samples. This related to how precipitates forced the dislocations to climb over particles and resulted in an increase in the creep resistance at least during the primary creep.

**Figure 10:**
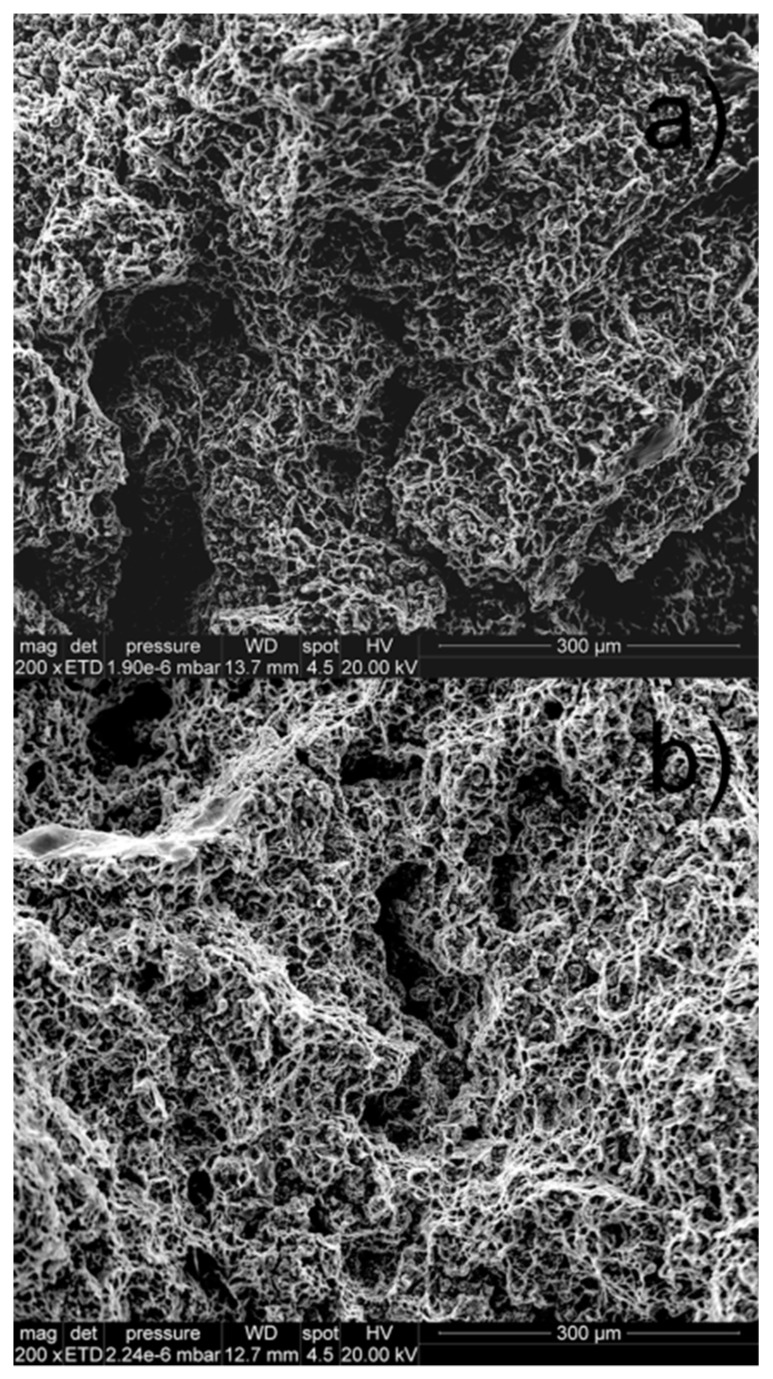
Fracture surfaces after creep. (**a**) Sample at 70 MPa; (**b**) pre-treated sample at 70 MPa.

Finally, a fractographic analysis of the fracture surfaces after creep is shown in Figure 10, where microvoid coalescence can be observed. The existence of pores is also visible. This porosity accelerated the strain rate during tertiary creep until fracture. 

## 4. Conclusions

The following conclusions were obtained:

(1) A short pre-treatment of 1 h at 160 °C stimulated the formation of continuous precipitation during creep in HPDC AZ samples. A plausible reason for this could be that small nuclei of β-phase were formed in the pre-treated samples and, when submitted to creep, the strains and temperatures enhanced the formation of continuous precipitates from these nuclei.
(2) The minimum creep rate that corresponded to the secondary creep region was lower for pre-treated samples than for the conventional samples. The higher stresses (70 and 65 MPa) did not permit a stable secondary creep region to be reached.
(3) The stress exponent, n, was higher for pre-treated samples and for both types of samples, treated and non-treated, is indicating that the mechanism of creep is dislocation climbing.
(4) The creep response of the pre-treated samples was better in the sense of having a lower strain rate at all stages of creep when the loads were less than 60 MPa.

## Figures and Tables

**Table 1:** Values of n coefficient and n_t_ true coefficient.

Samples	n	n_t_
Non-treated samples	4.8	4
Treated samples	5.6	4.8
Spigarelli et al. [14]	5	-
Vagarali & Langdon [16], Mg-0.8%Al, T4	3.6	-
Ishikawa &Watanabe [17], AZ31 with T4	5–7	-
Kaveh et al. [18], as-cast AZ91	5.6	-
